# The Cellular Immune Mechanism after Transfer of Chemically Extracted Acellular Nerve Xenografts

**DOI:** 10.1371/journal.pone.0068806

**Published:** 2013-07-17

**Authors:** Wei Li, Zhiwei Jia, Shunxin Zhang, Xingshi Lin, Ruojia Yang, Qing He, Dike Ruan

**Affiliations:** 1 Department of Orthopaedics, Navy General Hospital, Beijing, China; 2 South Clinical Department, PLA General Hospital, Beijing, China; 3 Department of Immunity, PLA General Hospital, Beijing, China; Glasgow University, United Kingdom

## Abstract

Severe peripheral nerve defect by injuries causing functional loss require nerve grafting. Autograft has limitations for clinical use because it results in the creation of a new nerve injury and the generation of donor site morbidity. Based on these limitations, nerve allografts and xenografts provide a readily accessible alternative strategy. The aim of the present study was to observe the immune mechanism underlying the rejection of chemically extracted acellular nerve xenografts, and further evaluate immunogenicity of chemically treated acellular nerve grafts for clinical applications. A total of 160 BALB/c mice were randomly divided into a negative contrast group (NC, 40 mice), a fresh autograft group (AG, 40 mice), a fresh xenogeneic nerve group (FXN, 40 mice) and a chemically extracted acellular xenogeneic nerve group (CEXN, 40 mice). Various types of nerve grafts were implanted into the thigh muscle of BALB/C mice in the corresponding groups. At 3, 7, 14 and 28 days post-operation, the mice (10 mice from each group) were sacrificed and their spleens were extracted. The spleens were ground into paste. The erythrocytes and other cells were lysed using distilled water and the T lymphocytes were collected. Fluorescein isothiocyanate (FITC) -labeled monoclonal antibodies (CD3, CD4, CD8, CD25, IL-2, IFN-γ and TNF-α) were then added to the solution. The Fluorescence Activated Cell Sorting (FACS) was used to determine the positivity rate of the cells combined with the monoclonal antibodies above. No significant statistical differences were observed between the CEXN, NC and AG groups, so that no obvious immune rejections were observed among the chemically extracted acellular nerve xenografts.

## Introduction

Peripheral nerve repair is one of the challenges of clinical practice. If transection injuries are not surgically repaired, the patient can be subjected to lifelong disability, pain, and impaired quality of life [1.2]. The surgical goal of nerve reconstruction is to achieve a tension-free repair [3]. Severe peripheral nerve defect require nerve grafting if injuries are not surgically direct repaired. Autografts are recognized as the gold standard for nerve grafting [4]. However, it has limitations for clinical use because it results in the creation of a new nerve injury, the generation of donor site morbidity, and increased operative time [5]. Based on these limitations, nerve grafts including allografts and xenografts provide a readily accessible alternative strategy.

Nerve allografts have been used to overcome the limitations of autografts, but their use is impaired by host immune rejection [6]. It has been known for years that after discontinuation of immunosuppressive agents, heterogeneous nerve-transplanted Schwann cells exhibit rejection [7]. Subsequent studies have confirmed the immunogenicity of Schwann cells, which show transplant immune rejection [8.9]. Cellular immune responses play a critical role in nerve graft rejection [10].The majority of the antigens in a transplanted nerve are associated with cells (such as Schwann cell), followed by the myelin sheath; the levels of antigens of the collagen and extracellular matrix, including Schwann cell basal lamina, are very low [11].

Schwann cells have the ability to synthesize, transfer and express major histocompatibility complex class II (MHC II) antigens and release cytokines that induce T cells to differentiate [11]. Many Schwann cell surfaces in vivo can express MHC II [12]. When adult Schwann cells are co-cultured with sensitive T cells, they express MHC II antigens; this indicates that Schwann cells can function as antigen-presenting cells because they can present antigens to antigen-specific T cell lines. [13]. Subsequently, the ability of Schwann cells in vivo to express MHC class II molecules was investigated [14], which strengthened the possibility that Schwann cells can function as accessory cells in the initiation or augmentation of T cell-mediated immune responses. Besides Schwann cells, another research showed that endothelial cells are also antigen-presenting cells in peripheral nerve [15]. A certain amount of MHC II expression is present in endothelial cells subjected to immune rejection [16].

Based on immune rejection, there were many research about the methods for the treatment of removal of antigen in peripheral allogeneic nerve, such as deep-frozen nerve grafts [17], freezing-drying nerve grafts [18], frozen-irradiated nerve grafts [19] and freezing-thawing nerve grafts [20]. They are more mature and simpler to apply. However, their effects are also unreliable because they can not remove Schwann cells and myelin sheaths throughout [11.21]. It was encouraging when chemical extraction was used to treat allogeneic nerve grafts [22]. Recent researchs showed that the main histocompatibility complex antigens within the aforementioned neural stem and the myelin sheath can be effectively removed, greatly reducing immunogenicity and preventing rejection [23]; the neural tube membrane and the lamellar structure are retained, providing a promising therapeutic approach to promote axonal regeneration [24].

Although allograft nerves are generally considered significantly less antigenic after chemical treatment, the source is not very sufficient. Additionally, commercially available peripheral nerve allograft are widely used nowadays, but lots of patients can not afford the high price. Therefore, nerve xenografts maybe become another alternative. Though there are few literature on nerve xenograft [25], the aim of our present work is to evaluate immunogenicity of chemically treated acellular nerve xenograft, and confirm the safety of the application of acellular nerve graft. The cellular immune mechanism mainly about T-lymphocyte subsets were studied after chemically extracted acellular nerve xenografts were transplanted, as well as changes in activated T cells and intracellular cytokine expression.

## Materials and Methods

### 1. Animals

Up to 180 healthy 6-week-old male BALB/C mice (weighing 20 g) and 20 healthy adult male New Zealand rabbits (weighing 2.5–2.6 Kg) were used. 20 cases of these BALB/C mice (5 mice on each operation day) were used as donors of autologous nerve for further operation. All animals were purchased from the Experimental Animal Center of PLA General Hospital (Beijing, China). All animals were housed in a pathogen-free animal facility and maintained in accordance with Institutional Animal Care and Use Committee and national law guidelines on the care and use of laboratory animals. All surgical procedures and postoperative care of the animals were approved by the Institutional Animal Ethics Committee. The protocol was approved by the Committee on the Ethics of Animal Experiments of PLA General Hospital and Chinese PLA Postgraduate Medical College(2011-A-302).

### 2. Preparation of Transplanted Nerves

**Figure 1:**
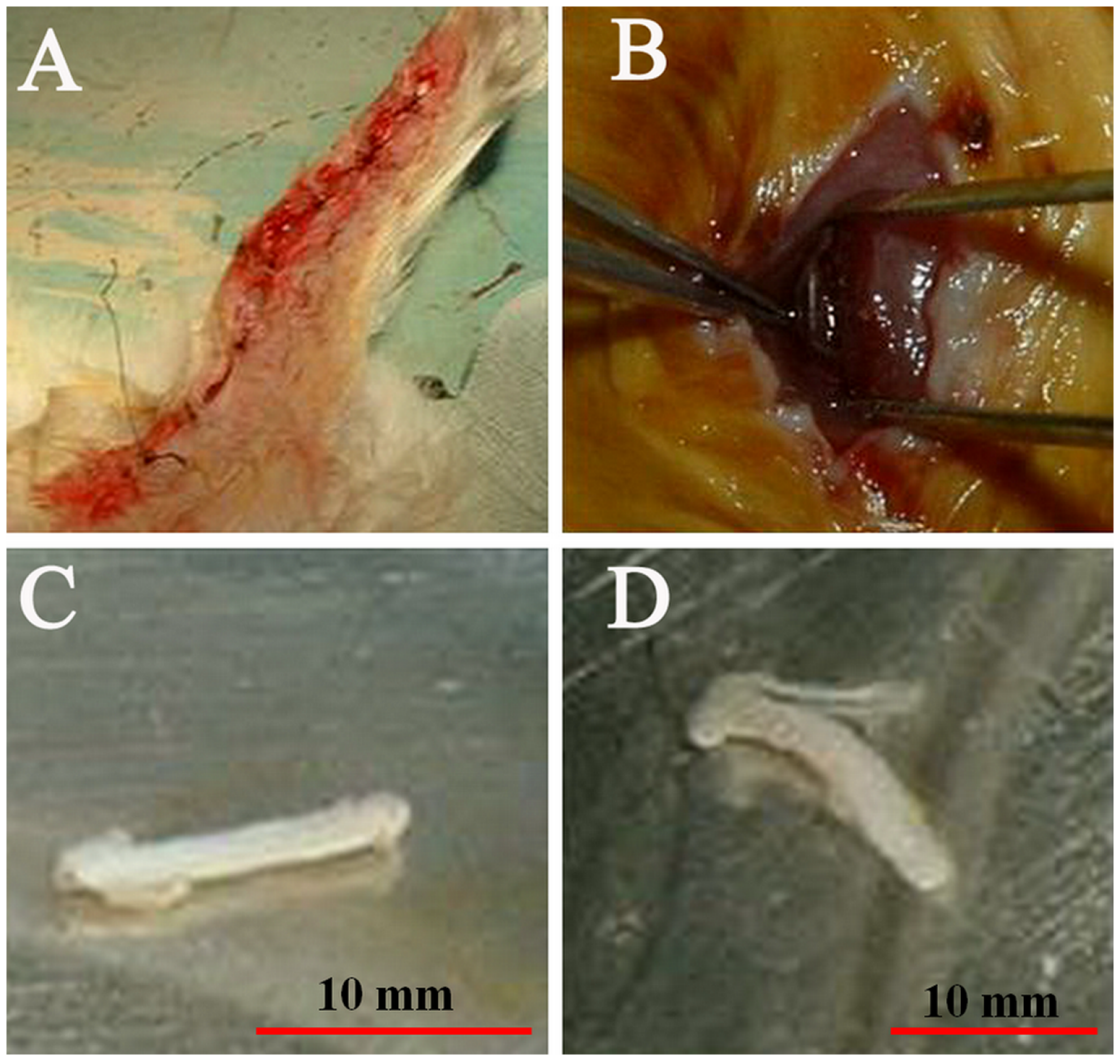
Nerves were harvested and processed for each group. (**A**) The skin incision was closed after ulner nerves were bilaterally harvested from the rabbits for further use in the chemically extracted acellular xenogeneic nerve group (CEXN group); (**B**) Sciatic nerves were harvested from BALB/c mice for immediate use in the fresh autograft group (AG group); (**C**) Fresh xenogeneic ulner nerves (0.3 mm in diameter and 12 mm long ) were harvested from New Zealand rabbits for immediate use in fresh xenogeneic nerve group (FXN group); (**D**) Xenogeneic ulner nerves (0.3 mm in diameter and 12 mm long ) were treated by chemical extraction for further use in the CEXN group.

10 healthy adult male New Zealand rabbits were used as donors of nerve xenograft. Others were used as donors of fresh xenogeneic nerve for further operative treatment. Male New Zealand rabbits were anesthetized with Methoxyflurane (induction: 3% in 100% O_2_; maintenance, 1% to 1.5%). Onset of anaesthesia was checked with loss of palpebral reflex and sensation pin prick over corresponding operative areas. Surgery commenced in 10 mins. Ulner nerves, 0.3 mm in diameter and 12 mm long, were bilaterally harvested from the rabbits (Fig. 1A), each donor provided 4 nerves refer to the standard above. The animals were sacrificed by air injection (20 ml) via auricular vein. The nerves were treated by using the Sondell method [22] for nerve chemical extraction, and then placed in sterile phosphate-buffered saline solution and stored at 4°C.

### 3. Animal Models

160 BALB/C mice were randomly divided into 4 groups (n = 40) as follows: NC, negative control group; AG, fresh autograft group; FXN, fresh xenogeneic nerve group; and CEXN, chemically extracted acellular xenogeneic nerve group. Male BALB/C mice were anesthetized with Methoxyflurane (induction: 3% in 100% O2; maintenance, 1% to 1.5%). Onset of anaesthesia was checked with loss of palpebral reflex and sensation pin prick over corresponding operative areas. Surgery commenced in 10 mins. The transplanted nerve that corresponds to each group was embedded into the muscle gap. The negative control group served as the control (mice had only operative treatment but no nerve grafts were embedded into muscle gap of the thigh); In the AG group, fresh sciatic nerves 0.3 mm in diameter and 12 mm long that harvested (Fig. 1B) on the operation day from previous BALB/c mice were transplanted, then the donors were sacrificed by cervical dislocation at each time point; Fresh ulner nerves (Fig. 1C) 0.3 mm in diameter and 12 mm long that harvested on the operation day from New Zealand rabbits, were transplanted in the FXN group, then the donors were sacrificed by air injection (20 ml) via auricular vein at each time point; Chemically extracted acellular ulner nerves (Fig. 1D) from New Zealand rabbits previous were transplanted in the CEXN group. All mice were randomly assigned and the nerves were transplanted within 1 day.

### 4. Experimental Index

Ten mice in each group (4 groups, all 40 mice) were sacrificed at each time point (on 3^rd^, 7^th^, 14^th^ and 28^th^ day) by cervical dislocation. After that, the abdominal operative region was soaked in 75% ethanol for local disinfection. Then the spleen was cleaned, placed in a Petri dish and washed with normal saline (NS; 0.9% saline solution). Up to 5 ml of NS was added to each Petri dish and a 200-mesh stainless steel sieve was subsequently immersed. The spleens of the mice were placed on the steel sieve and then ground with the plunger of a 5-ml syringe. The spleen cell suspension was placed in a 50-ml glass centrifuge tube (Beckman Inc., USA) and centrifuged at 1500 rpm for 8 mins. The supernatant was aspirated, the sediment was loosened. After that, 20 ml of injection water was added and mixed rapidly for 15 mins. This was followed by 2 ml of 10% sodium chloride solution, and mixed with small amounts of 0.9% NS. The mixture was centrifuged at 1,500 rpm for 8 mins. The resulting pellet was then rinsed with 0.9% NS and then centrifuged for another 8 mins. A small amount of lymphocyte suspension was taken from the cell suspension liquid, dyed with trypan blue (Xiercheng Biotechnology Co., Beijing, China), placed on a plate and counted. Based on the count of each tube, the extracted cell suspension was added to each tube to adjust the lymphocytes to 1×10^6^/tube. Each Eppendorf tube was marked and then centrifuged at 3,000 rpm for 3 mins. Fluorescein isothiocyanate (FITC) -labeled monoclonal antibodies (5 µl each tube; CD3, CD4, CD8, CD25, IL-2, IFN-γ, TNF-α monoclonal antibodies) (BD Pharmingen, Franklin Lakes, NJ USA) were added to the corresponding labeled Eppendorf tubes and were mixed thoroughly; One was left as the negative control. Each tube was placed in the dark at 4°C for 30 mins. After that, the excess antibodies in each tube were washed by phosphate buffer saline (PBS) (Xiercheng Biotechnology Co., Beijing, China) and the solution was fixed with 2% paraformaldehyde for flow cytometry (BD FACScalibur, BD, USA). Up to 9,400–10,000 lymphocytes were counted in each labeled Eppendorf tube. The T-lymphocyte subsets (expressing CD4+, CD8+, CD3+ by FITC-labeled monoclonal antibodies, the antibodies were purchased from BD Pharmingen Co., Franklin Lakes, NJ USA), activated T cells (expressing CD25+ by FITC-labeled monoclonal antibodies, the antibodies were purchased from BD Pharmingen Co., Franklin Lakes, NJ USA) and the percentage of lymphocytes expressing IL-2, IFN-γ and TNF-α (by FITC-labeled monoclonal antibodies, the antibodies were purchased from BD Pharmingen Co., Franklin Lakes, NJ USA) were analyzed with CellQuestTM software.

### 5. Statistical Analysis

The data were analyzed using Stata10.0 statistical software for single-factor ANOVA. Pairwise comparison between each group was analyzed by the Bonferroni method and mean (SD) represents the average value. P<0.05 was considered statistically significant.

## Results

### 1. General Data

Four mice died because of anesthesia, intra-operative and post-operative bleeding during the experiments. The others survived. The mice gained consciousness 0.5–2.0 hours after the operation. They were less active and exhibited poor feeding within 12–36 hrs. The wound of the mice swelled within 24–48 hours after the surgery, but no bleeding and exudates were present and all the mice regained their normal gait after 3 days. We just evaluated their activity postoperative by sensation pinprick in extremities, the sensation of the animals with grafts were normal and there were no limitation of motion, and no other abnormal symptoms were observed.

### 2. Activated T Cells and T-lymphocyte Subsets

**Figure 2:**
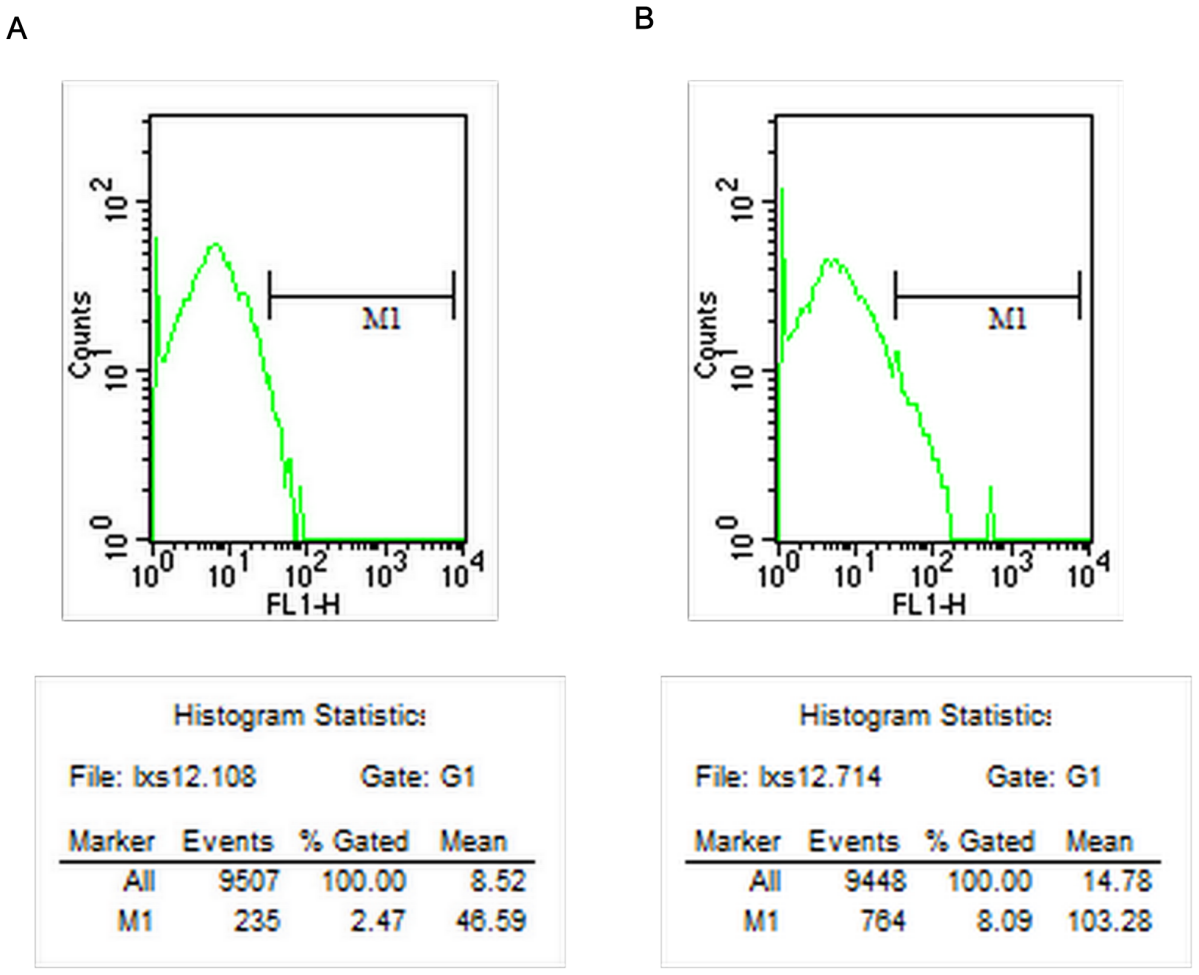
Counts of CD25+ T lymphocytes 14 days after surgery; (A) CEXN group (B) FXN group.

**Figure 3:**
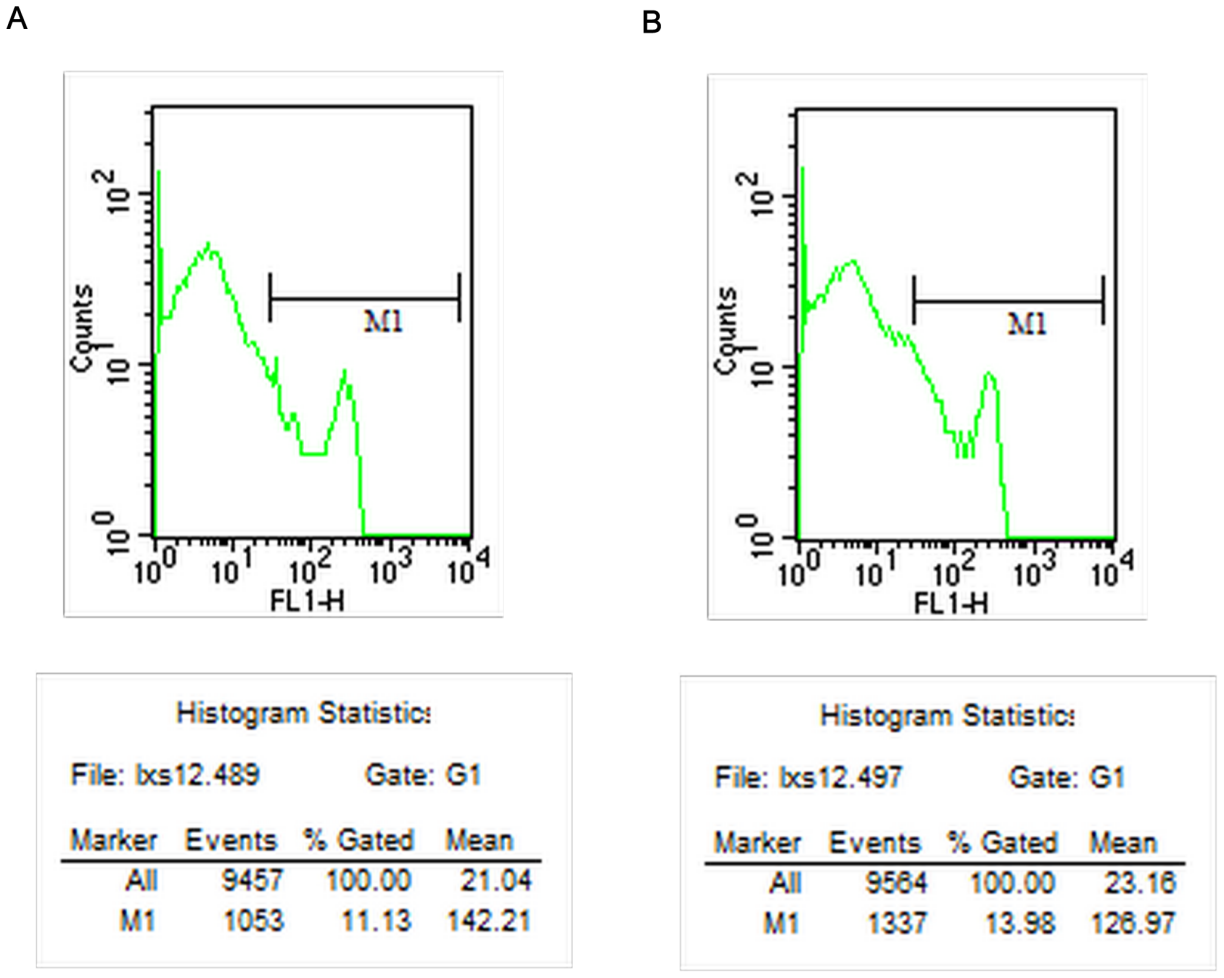
Counts of CD8+ T lymphocytes 14 days after surgery; (A) CEXN group (B) FXN group.

The percentages of T lymphocyte subsets and activated T lymphocytes detected by flow cytometry at different time points are shown in Tables 1–4. The counts of CD25+ T lymphocytes 14 days after surgery by flow cytometry in the CEXN group is shown in Fig. 2A, and the FXN group is shown in Fig. 2B. The counts of CD8+ T lymphocytes 14 days after surgery by flow cytometry in the CEXN group is shown in Fig. 3A, and the FXN group is shown in Fig. 3B. The results showed that in the FXN group, the T cell expression of CD25+,CD3+, CD4+ and CD8+ at 7 to 28 days were significantly higher and statistically significant (P<0.05; P<0.01) difference compared with the other three groups. However, in the CEXN group, the all above T cells expression at all time points showed no significant difference compared with the NC group and the AG group.

**Table 1:** Comparison of T lymphocytes and activated T lymphocytes 3 days after surgery in all the experimental groups (single-factor ANOVA).

	NC	AG	FXN	CEXN	F-value	P-value
(%)CD3+	38.61(2.02)	39.32(2.59)	39.55(2.19)	40.08(2.81)	0.62	0.61
(%)CD4+	20.59(2.32)	20.50(1.57)	21.21(2.67)	20.75(1.61)	0.22	0.86
(%)CD8+	10.86(1.50)	10.46(1.46)	10.26(1.99)	11.10(1.24)	0.52	0.67
(%)CD25+	2.46(0.24)	2.39(0.38)	2.25(0.33)	2.54(0.26)	1.58	0.21

NC, negative control group; AG, fresh autograft group; FXN, fresh xenogeneic nerve group; CEXN, chemically extracted acellular xenogeneic nerve group. ANOVA showed no significant differences between each group. Values represent mean (SD).

**Table 2:** Comparison of T lymphocytes and activated T lymphocytes 7 days after surgery in all the experimental groups (single-factor ANOVA).

	NC	AG	FXN	CEXN	F-value	P-value
(%)CD3+	38.57(2.50)	39.94(2.06)	45.87(3.92)a	39.08(2.94)	12.71	<0.01
(%)CD4+	19.74(1.83)	21.22(1.37)	23.90(2.27)b,c	20.58(1.73)	9.30	<0.01
(%)CD8+	10.38(1.18)	10.30(1.19)	12.94(1.33)a	10.87(1.30)	9.69	<0.01
(%)CD25+	2.58(0.27)	2.40(0.28)	5.73(0.40)a	2.65(0.30)	242.78	<0.01

ANOVA showed significant differences within groups (P<0.01). For pairwise comparison between each group, the Bonferroni method was used, Values represent mean (SD). **a:** FXN group compared with NC group (P<0.01), AG group (P<0.01), and CEXN group (P<0.01). **b:** FXN group compared with NC group (P<0.01), FXN group (P<0.01), and CEXN group (P<0.01). **c:** FXN group compared with AG group (P<0.05).

**Table 3:** Comparison of T lymphocytes and activated T lymphocytes 14 days after surgery in all the experimental groups (single-factor ANOVA).

	NC	AG	FXN	CEXN	F-value	P-value
(%)CD3+	39.95(1.53)	41.41(1.54)	49.22(2.85)a	40.03(2.91)	36.83	<0.01
(%)CD4+	20.30(1.35)	20.81(1.12)	25.41(2.03)a	20.32(1.30)	27.68	<0.01
(%)CD8+	11.14(1.32)	10.87(1.09)	13.85(1.56)a	11.43(1.13)	11.27	<0.01
(%)CD25+	2.73(0.31)	2.60(0.28)	7.93(0.64)a	2.74(0.23)	433.22	<0.01

ANOVA showed significant differences within groups (P<0.01). For pairwise comparison between each group, the Bonferroni method was used, Values represent mean (SD). **a:** FXN group compared with NC group (P<0.01), AG group (P<0.01), and CEXN group (P<0.01).

**Table 4:** Comparison of T lymphocytes and activated T lymphocytes 28 days after surgery in all the experimental groups (single-factor ANOVA).

	NC	AG	FXN	CEXN	F-value	P-value
(%)CD3+	37.77(1.71)	39.04(1.88)	46.52(2.20)a	38.65(2.38)	38.16	<0.01
(%)CD4+	18.63(1.87)	18.90(1.92)	22.96(2.24)b,c	19.83(2.87)	7.05	<0.01
(%)CD8+	10.74(1.26)	10.13(0.86)	12.80(1.21)a	10.68(1.16)	10.71	<0.01
(%)CD25+	2.81(0.30)	2.67(0.32)	7.39(0.55)a	2.91(0.29)	363.57	<0.01

ANOVA showed significant differences within groups (P<0.01). For pairwise comparison between each group, the Bonferroni method was used, Values represent mean (SD). **a:** FXN group compared with NC group (P<0.01), AG group (P<0.01), and CEXN group (P<0.01). **b:** FXN group compared with NC group (P<0.01), and AG group (P<0.01). **c:** FXN group compared with CEXN group (P<0.05).

### 3. Intracellular Cytokines

**Figure 4:**
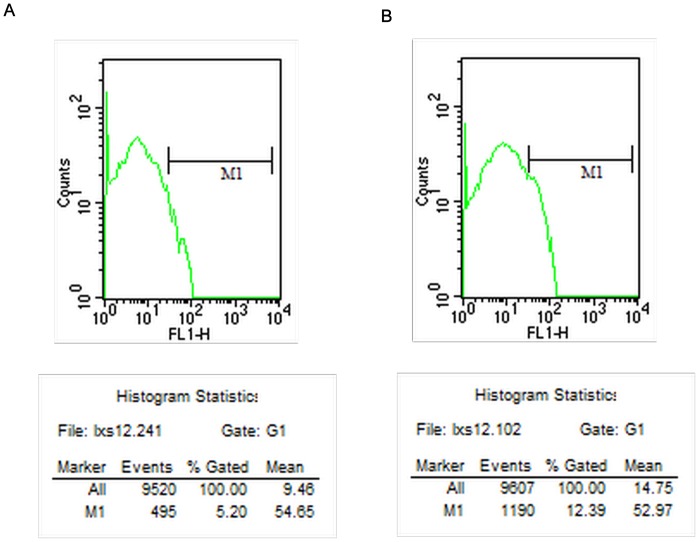
Counts of cells expressing IFN-ã 14 days after surgery; (A) CEXN group (B) FXN group.

**Figure 5:**
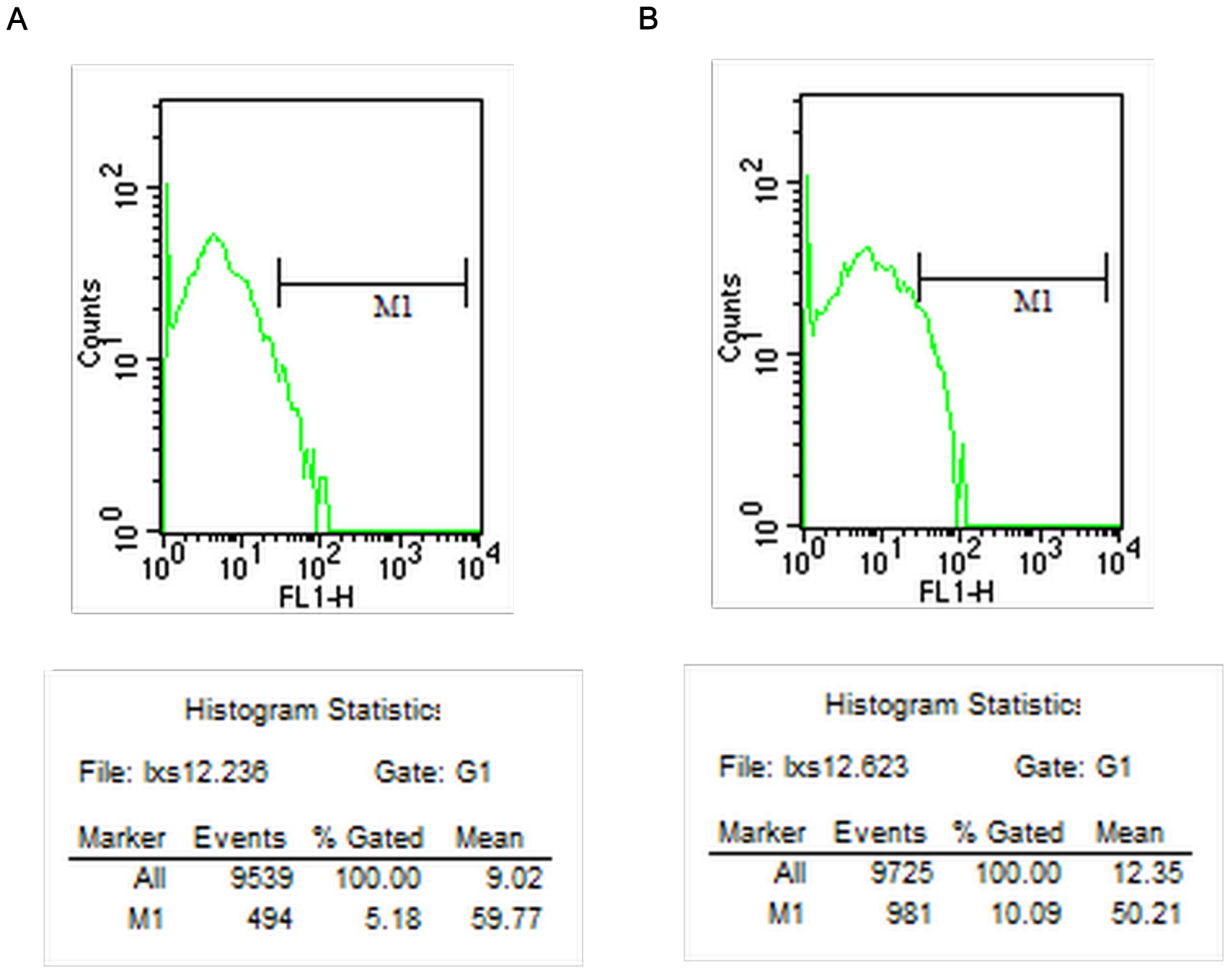
Counts of cells expressing IL-2 14 days after surgery; (A) CEXN group (B) FXN group.

The intracellular cytokine expression levels detected by flow cytometry at different time points are shown in Tables 5–8. The counts of cells expressing IFN-γ 14 days after surgery by flow cytometry in the CEXN group is shown in Fig. 4A, and the FXN group is shown in Fig. 4B. The counts of cells expressing IL-2 14 days after surgery by flow cytometry in the CEXN group is shown in Fig. 5A, and the FXN group is shown in Fig. 5B. The results showed that from 7 to 28 days, the IL-2, IFN-γ and TNF-α expression in the FXN group were significantly higher and statistically significant (P<0.01) difference compared with the other three groups. In contrast, there were no statistically significant in all above cytokines expression in NC group, AG group and CEXN group at all time points.

**Table 5:** Comparison of intracellular cytokine expression in all experimental groups 3 days after surgery (single-factor ANOVA).

	NC	AG	FXN	CEXN	F-value	P-value
(%)IL-2	3.91(0.21)	3.96(0.17)	3.94(0.23)	3.87(0.21)	0.33	0.81
(%)IFN-γ	4.10(0.20)	4.15(0.29)	4.18(0.19)	4.13(0.29)	0.23	0.87
(%)TNF-α	3.67(0.19)	3.52(0.22)	3.57(0.20)	3.65(0.25)	0.95	0.43

ANOVA showed no significant differences between each group. Values represent mean (SD).

**Table 6:** Comparison of intracellular cytokine expression in all experimental groups 7 days after surgery (single-factor ANOVA).

	NC	AG	FXN	CEXN	F-value	P-value
(%)IL-2	3.96(0.24)	4.11(0.21)	7.15(0.31)a	4.09(0.17)	416.32	<0.01
(%)IFN-γ	4.08(0.29)	4.25(0.24)	9.24(0.55)a	4.34(0.27)	470.38	<0.01
(%)TNF-α	3.70(0.24)	3.85(0.29)	5.66(0.38)a	3.82(0.27)	93.65	<0.01

ANOVA showed significant differences within groups (P<0.01). For pairwise comparison between each group, the Bonferroni method was used, Values represent mean (SD). **a:** FXN group compared with NC group (P<0.01), AG group (P<0.01), and CEXN group (P<0.01).

**Table 7:** Comparison of intracellular cytokine expression in all experimental groups 14 days after surgery (single-factor ANOVA).

	NC	AG	FXN	CEXN	F-value	P-value
(%)IL-2	4.50(0.36)	4.68(0.35)	9.68(0.49)a	4.76(0.36)	409.78	<0.01
(%)IFN-γ	4.70(0.31)	4.84(0.29)	11.77(0.83)a	4.83(0.35)	493.38	<0.01
(%)TNF-α	4.03(0.21)	3.99(0.31)	6.32(0.42)a	4.09(0.24)	140.41	<0.01

ANOVA showed significant differences within groups (P<0.01). For pairwise comparison between each group, the Bonferroni method was used, Values represent mean (SD). **a:** FXN group compared with NC group (P<0.01), AG group (P<0.01), and CEXN group (P<0.01).

**Table 8:** Comparison of intracellular cytokine expression in all experimental groups 28 days after surgery (single-factor ANOVA).

	NC	AG	FXN	CEXN	F-value	P-value
(%)IL-2	4.23(0.28)	4.29(0.37)	7.79(0.65)a	4.40(0.36)	156.46	<0.01
(%)IFN-γ	4.42(0.37)	4.36(0.25)	9.97(0.82)a	4.41(0.32)	319.12	<0.01
(%)TNF-α	3.82(0.25)	3.69(0.32)	5.34(0.46)a	3.89(0.30)	51.97	<0.01

ANOVA showed significant differences within groups (P<0.01). For pairwise comparison between each group, the Bonferroni method was used, Values represent mean (SD). **a:** FXN group compared with NC group (P<0.01), AG group (P<0.01), and CEXN group (P<0.01).

## Discussion

Cellular immune mechanism plays a critical role in nerve graft rejection [11.26]. In the body, as we know, all mature T lymphocyte cells surfaces express the CD3+ antigen, and MHC II antigens on the surface of most nucleated cells have been identified as CD8+ [27]. The MHC II antigen are expressed in B lymphocytes, monocytes, macrophages, dendritic cells, vascular endothelial and ductal epithelial cells, which are all T lymphocyte antigen-presenting cells and could be identified by CD4+ T cells [28].

Acute rejection generally occurs within one week to six months after transplantation. The main immune response in allogeneic or xenogeneic nerve transplants is cell-mediated [29]. Compared with autografts and allografts, xenografts can induce even more stronger rejection [25.26]. Through direct or indirect activation of helper T cells, foreign antigens secrete cytokines, such as IL-2 to activate CTL. IFN-γ, TNF-α activates monocytes, whereas IL-1, IL-2 and IL-4 activate B cells into plasma cells to produce specific antibodies and then attack the target cells, tissues and organs, which causes vascular endothelial cell damage. A series of intracellular cytokines (such as IL-2 and IFN-γ) released by helper T cells may reflect the immune status and transplant rejection [30].

As we mentioned previously, effects of methods for removal of antigens from peripheral allogeneic nerve such as deep-frozen, freezing-drying, frozen-irradiated and freezing-thawing nerve grafts are unreliable because they can not remove Schwann cells and myelin sheaths throughout. Additionally, according to our own experience, some patients would rather choose autologous nerve grafts, even xenografts than allografts as their traditional ethical reasons, and lots of patients can not afford the high price of the commercially available peripheral nerve allograft. Actually, the main idea of this work is to evaluate immungenicity of chemically treated acellular nerve grafts, especially that of xenograft for further clinical application.

In this experiment, CD3+, CD4+ and CD8+ T cells expression in the CEXN group showed no significant change by flow cytometry at 3–14 days after transplantation. After that, at 28 days after transplantation, similar to the NC group and AG group, the expression in the CEXN group decreased slightly. In contrast, CD25+ T cells expression at each time point from 3 to 28 days increased slightly. However, the CD3+, CD4+, CD8+ and CD25+ T cells expression at all time points showed no significant difference compared with the NC group and the AG group. In the FXN group, the T cell expression of CD3+, CD4+ and CD8+ at 7 to 28 days were significantly higher, which peaked at day 14 and decreased at 28 days; the T cell expression of CD25+ in FXN group also increased at all time points from 7 to 28 days and reached a peak at day 14; and the expression of CD3+, CD4+, CD8+ and CD25+ T cell during the period of 7–28 days were statistically significant (P<0.05; P<0.01) difference compared with the other three groups. In contrast, the number of cells positive for IL-2, IFN-γ and TNF-α in the NC group, AG group and CEXN group increased slightly, as shown by flow cytometry during the period of 3–14 days and eventually decreased slightly at 28 days, and there were no statistically significant in cytokine (IL-2, IFN-γ and TNF-α) expression in these three groups at all time points. In the FXN group, the number of cells positive for IL-2, IFN-γ and TNF-α increased sharply from 7 to 14 days, which peaked at day 14 and eventually decreased at 28 days. From 7 to 28 days, the IL-2, IFN-γ and TNF-α expression in the FXN group were significantly higher (P<0.01) than those of NC group, AG group and CEXN group.

In conclusion, fresh peripheral nerve xenograft rejection after transplantation is an acute course. The rejection could be significantly decreased after chemical nerve extraction because it could effectively remove the MHC cells in the major nerve trunk, thereby reducing its immunogenicity and minimizing the risk of rejection. The use of chemical extraction to treat the cells significantly reduces antigenicity. Immunogenicity of the nerve xenograft after the chemical extraction treatment is equal to or close to that of autologous nerves and significantly lower than that of fresh nerve xenografts. These findings confirm the safety of the chemically extracted acellular peripheral nerve grafts for clinical application.
